# Variation in promiscuity and sexual selection drives avian rate of Faster‐Z evolution

**DOI:** 10.1111/mec.13113

**Published:** 2015-03-16

**Authors:** Alison E. Wright, Peter W. Harrison, Fabian Zimmer, Stephen H. Montgomery, Marie A. Pointer, Judith E. Mank

**Affiliations:** ^1^ Department of ZoologyEdward Grey InstituteUniversity of OxfordOxfordOX1 3PSUK; ^2^ Department of Genetics, Evolution and EnvironmentUniversity College LondonLondonWC1E 6BTUK

**Keywords:** effective population size, Faster‐Z evolution, genetic drift, sexual selection

## Abstract

Higher rates of coding sequence evolution have been observed on the Z chromosome relative to the autosomes across a wide range of species. However, despite a considerable body of theory, we lack empirical evidence explaining variation in the strength of the Faster‐Z Effect. To assess the magnitude and drivers of Faster‐Z Evolution, we assembled six *de novo* transcriptomes, spanning 90 million years of avian evolution. Our analysis combines expression, sequence and polymorphism data with measures of sperm competition and promiscuity. In doing so, we present the first empirical evidence demonstrating the positive relationship between Faster‐Z Effect and measures of promiscuity, and therefore variance in male mating success. Our results from multiple lines of evidence indicate that selection is less effective on the Z chromosome, particularly in promiscuous species, and that Faster‐Z Evolution in birds is due primarily to genetic drift. Our results reveal the power of mating system and sexual selection in shaping broad patterns in genome evolution.

## Introduction

Sex chromosomes are subject to unique evolutionary forces as a result of their unusual pattern of inheritance (Charlesworth *et al*. 1987; Vicoso & Charlesworth 2009; Connallon *et al*. 2012). The magnitude of selection, genetic drift and recombination are all predicted to differ between the sex chromosomes and autosomes (Rice 1984; Kirkpatrick & Hall 2004a; Mank *et al*. 2010a; Meisel & Connallon 2013) and studies contrasting the evolution of sex‐linked to autosomal genes can shed light on the fundamental evolutionary forces acting across the genome as a whole.

Faster rates of coding sequence evolution have been observed on the Z and X chromosomes relative to the autosomes across a wide range of species (recently reviewed by Meisel & Connallon 2013), and Faster‐X and Faster‐Z Effects appear to be a common feature of sex chromosome evolution. However, despite elevated rates of evolution for both X‐linked and Z‐linked genes, the underlying causes of Faster‐X and Faster‐Z Evolution are predicted to differ (Vicoso & Charlesworth 2009; Meisel & Connallon 2013).

**Figure 1:**
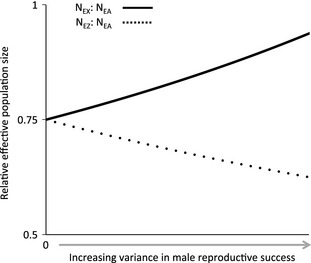
Relationship between effective population size (*N*_E_) and variance in male reproductive success. Schematic outlining the predicted relationship between variance in male reproductive success and relative *N*_EZ_ and *N*_EX_. When variance in reproductive success is the same in males and females, under monogamy, both *N*_EZ_ and *N*_EX_ = ¾ *N*_EA_. As variance in male mating success increases, *N*_EZ_ < ¾ *N*_EA_ and *N*_EX_ > ¾ *N*_EA_.

The effective population size of X and Z chromosomes (*N*_EX_ and *N*_EZ_) is ¾ that of the autosomes (*N*_EA_) when there is no difference in the variance of male and female reproductive success, such as in strictly monogamous breeding systems (Charlesworth *et al*. 1987). However, many forms of sexual selection cause elevated variance in male reproductive success (Andersson 1994), which reduces *N*_EZ_/*N*_EA_, and in extreme cases where a single male monopolizes the reproductive output of many females, *N*_EZ_ approaches ½ *N*_EA_ (Vicoso & Charlesworth 2009; Wright & Mank 2013) (Fig. 1). Correspondingly, genetic drift and fixation of weakly deleterious mutations is greater on the Z chromosome (Charlesworth 2009), and we predict a Faster‐Z Effect largely due to neutral, nonadaptive processes. Empirical evidence in birds and snakes is consistent with this nonadaptive and neutral explanation of Faster‐Z (Mank *et al*. 2010b; Corl & Ellegren 2012; Vicoso *et al*. 2013a); however, silk moths may present a recent exception (Sackton *et al*. 2014). It is worth noting that a major factor determining the relative contribution of nonadaptive and adaptive drivers of Faster‐Z is overall effective population size (Meisel & Connallon 2013). Overall *N*_E_ mediates the distribution of fitness effects, and specifically, we expect the efficacy of selection and adaptive component of Faster‐Z to be weaker in populations with smaller *N*_E_ (Kimura & Ohta 1971).

The opposite relationship between male mating success and relative *N*_EX_ is predicted in male heterogametic systems (Laporte & Charlesworth 2002; Vicoso & Charlesworth 2009; Wright & Mank 2013). Increasing variance in male reproductive success results in *N*_EX_/*N*_EA_ > ¾, and *N*_EX_/*N*_EA_ may approach 1 in extreme cases (Fig. 1). Correspondingly, the higher ratio of *N*_EX_/*N*_EA_ is expected to decrease the effect of genetic drift in Faster‐X Evolution. Elevated rates of evolution on X chromosomes are therefore more often thought to be the product of increased efficacy of selection acting on recessive X‐linked alleles in the heterogametic sex, thereby increasing the rate of fixation of beneficial alleles relative to the autosomes. Consistent with adaptive Faster‐X Evolution, signatures of positive selection have been uncovered on the X chromosome of mammals and *Drosophila* (Thornton & Long 2005; Baines *et al*. 2008; Hvilsom *et al*. 2012; Langley *et al*. 2012).

**Figure 2:**
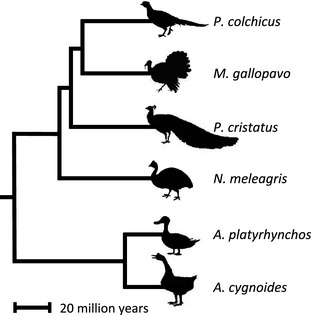
Phylogenetic relationship of the Galloanserae species in this study.

A key prediction is that the magnitude of Faster‐Z Evolution can be explained by variation in the effective population size of the sex chromosomes relative to the autosomes driven by sexual selection (Vicoso & Charlesworth 2009). Here, we explicitly test this prediction in the Galloanserae, a clade of birds spanning 90 million years (Fig. 2), for which there is extensive variation in mating system (Moller 1988, 1991; Birkhead & Petrie 1995). Using *de novo* transcriptomes for six Galloanserae species, we measured sequence divergence, polymorphism and expression and combined these molecular data with phenotypic measures of mating system to explore the nature of Faster‐Z Evolution. Our results build on previous findings to reveal the dominant role nonadaptive processes play in Faster‐Z. Furthermore, we uncover a positive association between Faster‐Z and measures of sperm competition, a widely used indicator of the strength of postcopulatory sexual selection (Birkhead & Moller 1998). Our results suggest that variation in male mating success drives Z‐linked divergence, and present the first empirical evidence in support of the considerable body of theory (Charlesworth *et al*. 1987; Vicoso & Charlesworth 2009) outlining the relationship between sexual selection and sex chromosome evolution.

## Materials and methods

### *De novo* transcriptome assembly

RNA‐Seq data were obtained from captive populations of the following Galloanserae species at the start of their first breeding season; *Anas platyrhynchos* (mallard duck), *Meleagris gallopavo* (wild turkey)*, Phasianus colchicus* (common pheasant)*, Numida meleagris* (helmeted guinea fowl)*, Pavo cristatus* (Indian peafowl) *and Anser cygnoides* (swan goose) (Fig. 2). Samples were collected with permission from institutional ethical review committees and in accordance with national guidelines. The left gonad and spleen were dissected separately from five males and five females of each species. The exceptions were *P. colchicus*, where six male gonad and spleen samples were collected, and *M. gallopavo*, where four male and two female spleens were collected. Samples were homogenzied and stored in RNA later until preparation. We used the Animal Tissue RNA Kit (Qiagen) to extract RNA, and the samples were prepared and barcoded at The Wellcome Trust Centre for Human Genetics, University of Oxford using Illumina's Multiplexing Sample Preparation Oligonucleotide Kit with an insert size of 280 bp. RNA was sequenced on an Illumina HiSeq 2000 resulting in on average 26 million 100 bp paired‐end reads per sample (Tables S1 and S2, Supporting Information).

The data were quality assessed using FastQC v0.10.1 (www.bioinformatics.babraham.ac.uk/projects/fastqc) and filtered using Trimmomatic v0.22 (Lohse *et al*. 2012). Specifically, we removed reads containing adaptor sequences and trimmed reads if the sliding window average Phred score over four bases was <15 or if the leading/trailing bases had a Phred score <4. Reads were removed post filtering if either read pair was <25 bases in length. We constructed *de novo* transcriptome assemblies for each species using trinity with default parameters (Grabherr *et al*. 2011). We separately mapped back all of the reads from each sample to the Trinity contigs using rsem v1.1.21 with default parameters (Li & Dewey 2011) to obtain expression levels. We applied a minimum expression filter of 2 reads per kilobase per million mapped reads (RPKM) requiring that each contig has expression above unlogged 2 RPKM in at least half of any of the tissues from either sex. For each Trinity contig cluster, the isoform with the highest expression level was selected for further analysis. We removed rRNA transcripts using *G. gallus* known sequences. This generated 37453 contigs for *A. platyrhynchos*, 50817 for *M. gallopavo,* 56090 for *P. colchicus,* 45535 for *N. meleagris,* 56604 for *P. cristatus* and 44144 for *A. cygnoides*.

### Identification of Galloanserae orthogroups

*G. gallus* (Galgal4/GCA_000002315.2) cDNA sequences were obtained from ensembl v73 (Flicek *et al*. 2013), and the longest transcript for each gene was identified. We determined orthology using reciprocal blastn v2.2.27+ (Altschul *et al*. 1990) with an *E*‐value cut‐off of 1 × 10^−10^ and minimum percentage identity of 30%. Reciprocal 1‐1 orthologs across all seven species (orthogroups) were identified using the highest blast score.

Avian chromosome structure is unusually stable, potentially due to a lack of active transposons (Toups *et al*. 2011), and major genomic rearrangements are infrequent (Stiglec *et al*. 2007). Synteny of the Z chromosome has previously been shown to be highly conserved across both extant birds (Vicoso *et al*. 2013b), as well as within the Galloanserae (Skinner *et al*. 2009). Chromosomal location was therefore assigned from *G. gallus* reciprocal orthologs.

### Estimating sequence divergence across orthogroups

To extract Galloanserae protein‐coding sequences*, G. gallus* (Galgal4/GCA_000002315.2) protein sequences were obtained from ensembl v73 (Flicek *et al*. 2013). For each orthogroup, each contig was translated into all potential reading frames and blasted against the orthologous *G. gallus* protein sequence using blastx. blastx outputs were used to determine coding frame, and protein‐coding sequences for each species were extracted. Protein‐coding sequences were defined as sequences starting with the amino acid M and terminating with a stop codon or end of the contig. Orthogroups with no blastx hits or a valid protein‐coding sequence were excluded.

Orthogroups were aligned with prank v121218 using the orthologous *Taeniopygia guttata* cDNA (taeGut3.2.4.75) as an outgroup and specifying the following guidetree (((*A. cygnoides*,* A. platyrhynchos*), (*N. meleagris*, (*P. cristatus*, (*M. gallopavo*,* P. colchicus*)))), *T. guttata*). Retrotransposons were removed with repeatmasker (v open‐4.0.3), and sequences with internal stop codons were also removed. swamp v0.9 (Harrison *et al*. 2014) with a cut‐off of 4 and window size of 15, and a minimum length of 75 bp was used to preprocess the data.

To obtain divergence estimates for each orthogroup, we used the branch model (model=2, nssites=0) in the codeml package in paml v4.7a (Yang 2007), using the specified phylogeny; ((*A. cygnoides*,* A. platyrhynchos*), (*N. meleagris*, (*P. cristatus*, (*M. gallopavo*,* P. colchicus*))), *T. guttata*). The branch model was used to calculate mean *d*_N_/*d*_S_ across all Galloanserae branches, excluding the *T. guttata* outgroup. We will refer to this as the Galloanserae analysis. We also used the branch model to calculate mean *d*_N_/*d*_S_ for each of the six Galloanserae species separately. Specifically, for each species, we calculated mean *d*_N_/*d*_S_ from the terminal tip to the Galloanserae common ancestor. We will refer to this as the species‐specific analysis. This approach ensures that the branch length over which *d*_N_/*d*_S_ is calculated is identical for each species and therefore prevents interspecific variation in branch length biasing our conclusions (Montgomery *et al*. 2011). As mutational saturation and double hits can lead to inaccurate divergence estimates (Axelsson *et al*. 2008), orthogroups were excluded if tree length *d*_S_ >2 across all branches.

### Using sequence divergence to estimate the Faster‐Z Effect

The avian genome exhibits considerable karyotypic variation in chromosome size. Therefore, mean *d*_N_, *d*_S_ and *d*_N_/*d*_S_ were calculated separately for all autosomes, autosomes 1–10, microchromosomes and the Z chromosome. Microchromosomes exhibit an elevated recombination rate, greater gene density and GC content, all of which have been shown to impact the nature and efficacy of selection (Burt 2002; Ellegren 2013). The fairest measure of Faster‐Z Evolution is therefore to contrast divergence between the Z chromosome and similar‐sized autosomes 1–10 (Mank *et al*. 2010b).

For each genomic category, mean *d*_N_ and mean *d*_S_ were calculated as the sum of the number of substitutions across all contigs in a given category divided by the number of sites (*d*_N_ = sum *D*_N_/sum N, *d*_S_ = sum *D*_S_/sum S, where *D*_N/S_ is an estimate of the number of nonsynonymous/synonymous substitutions and N/S is the number of nonsynonymous/synonymous sites). This approach avoids the problems of infinitely high *d*_N_/*d*_S_ estimates arising from contigs with extremely low *d*_S_ (Mank *et al*. 2007a, 2010b) and prevents disproportionate weighting of shorter contigs.

Bootstrapping with 1000 repetitions was used to generate 95% confidence intervals, and significant differences between genomic categories were determined from 1000 permutation tests. One‐tailed *P*‐values are reported because we specifically test whether *d*_N_, *d*_S_ and *d*_N_/*d*_S_ are significantly higher for Z‐linked contigs vs. autosomal contigs. Mean Z‐linked and autosomal *d*_N_, *d*_S_ and *d*_N_/*d*_S_ values were calculated for the whole Galloanserae (Galloanserae analysis) and for each of the six species (species‐specific analysis). Faster‐Z Effect was calculated as *d*_NZ_/*d*_SZ_: *d*_NA_/*d*_SA_.

### Testing the relationship between sexual selection and Faster‐Z Effect

To test the hypothesis that the magnitude of Faster‐Z increases with increased variance in male reproductive success, we performed phylogenetically controlled regression analyses between Faster‐Z (*d*_NZ_/*d*_SZ_: *d*_NA_/*d*_SA_) and relative *N*_EZ_ for each Galloanserae species and two measures of female promiscuity. The intensity of sperm competition, a widely used proxy for the magnitude of postcopulatory sexual selection and therefore variance in male reproductive success, is strongly predicted by relative testes weight and sperm number (Moller 1991; Moller & Briskie 1995; Birkhead & Moller 1998). These measures are also frequently used to test genotype–phenotype hypotheses (e.g. Dorus *et al*. 2004; Ramm *et al*. 2008). Residual testes weight was calculated using the following equation describing the linear relationship between log testes weight and body weight across a large number of birds (Pitcher *et al*. 2005): log_2_[testes mass(g)] = −1.56 + 0.61 log_2_ [body mass(g)] (Moller 1988, 1991; Birkhead & Petrie 1995). For all six species in this study, relative testes weight was less than expected given body weight. Log sperm number (10^6) has been measured in previous studies (Moller 1988, 1991; Birkhead & Petrie 1995). Estimates for body weight and sperm number were not available for *A. cygnoides* and therefore *A. anser* estimates were used instead, as these species are closely related (Ruokonen *et al*. 2000) and both exhibit strictly monogamous mating systems.

These analyses were performed using phylogenetic generalized least squares models (PGLS) in bayestraits v2‐beta (Pagel 1999; Pagel *et al*. 2004) with maximum likelihood and 1000 runs for each analysis. PGLS corrects for phylogenetic nonindependence. Phylogenies were obtained from birdtree.org using the Ericson data set. For each regression analysis, mean *r* ^2^ and mean *t*‐value (mean regression coefficient/mean standard error) were calculated. A one‐tailed *t*‐test with four degrees of freedom was used to determine whether the slope was significantly >0.

Differences in the rate of male‐biased mutation across the six species could contribute to variation in Faster‐Z Effect because the Z chromosome is more often present in males than the autosomes (Kirkpatrick & Hall 2004a). We explicitly tested for significant differences in mean Z‐linked *d*_S_ across the six species using permutation tests with 1000 replicates to verify that were no underlying differences in mutation rate.

### Tests of positive selection using sequence data

To test for signatures of positive selection acting at a subset of sites, we used the site models in the codeml package in paml v4.7a (Yang 2007). These models allow *d*_N_/*d*_S_ to vary among sites but not across lineages. To test for positive selection, we compared likelihoods from two models; M1a (Nearly neutral, model=0, nssites=1) and M2a (Positive selection, model=0, nssites=2). Under model M1a, sites can fall into one of two categories (purifying selection *d*_N_/*d*_S_ <1 and neutral evolution *d*_N_/*d*_S_ = 1), whereas there is an additional category under model M2a (positive selection *d*_N_/*d*_S_ >1). The following phylogeny was specified; ((*A. cygnoides*,* A. platyrhynchos*), (*N. meleagris*, (*P. cristatus*, (*M. gallopavo*,* P. colchicus*))), *T. guttata*).

### Tests of positive selection using polymorphism data

We tested for deviations from neutrality using polymorphism data. Polymorphism data was obtained by first mapping RNA‐seq reads to orthogroups using the two‐pass alignment method of the star aligner with default parameters (Dobin *et al*. 2013). SNPs were called using varscan v2.3.6 (Koboldt *et al*. 2009, 2012) and samtools (Li *et al*. 2009) following the recommendations of Quinn *et al*. 2013 (Quinn *et al*. 2013). Only uniquely mapping reads were used to call SNPs. samtools was run with probabilistic alignment disabled and a maximum read depth of 10 000 000. varscan mpileup2snp was run with a minimum coverage of 2, a minimum average quality of 20, with the strand filter, *P*‐value of 1, a minimum variant allele frequency threshold of 1E‐1 and a minimum frequency to call homozygote of 0.85. SNPs were required to have a minor allele frequency >0.15 and to be from regions where at least 4 samples had a read depth >20 and have a Phred quality >20. Valid SNPs were matched to the reading frame to determine whether they were synonymous or nonsynonymous. Fixed sites were identified using the same quality and coverage thresholds used to call SNPs.

We explicitly tested whether our power to identify SNPs is equal across the Z and autosomes, despite differences in sequencing coverage. We generated random diploid populations of individuals with varying minor allele frequencies. From these populations, we sampled 20 (autosomal) and 15 (Z‐linked) alleles separately 1000 times without replacement and for each sample determined the presence or absence of polymorphism. At a minor allele frequency of 0.15%, the false‐negative rate for both the autosomes and Z chromosome was very low (autosomes = 0.023, Z chromosome = 0.068), although marginally lower for the autosomes. We also repeated analyses using a minor allele frequency threshold of 25% (false‐negative rate autosomes = 0.001, Z chromosome = 0.009); however, our power is limited at this threshold due to a large reduction in detectable SNPs (Tables S3 and S4, Supporting Information). Our conclusions were broadly comparable across both minor allele frequency thresholds.

For each species, mean nonsynonymous polymorphism (p_N_), synonymous polymorphism (p_S_) and p_N_/p_S_ were calculated separately for Z‐linked and autosomal 1–10 orthogroups. Specifically, mean polymorphism was calculated as the sum of the number of polymorphic sites across all contigs in a given genomic category divided by the number of sites (p_N_ = sum P_N_/sum N, p_S_ = sum P_S_/sum S where P_N/S_ is the number of nonsynonymous/synonymous polymorphic sites and N/S is the number of nonsynonymous/synonymous sites). Faster‐Z was calculated as p_NZ_/p_SZ_: p_NA_/p_SA_. Bootstrapping with 1000 repetitions was used to generate 95% confidence intervals, and significance differences between genomic categories were determined from 1000 permutation tests.

For each species, we used the McDonald–Kreitman test (McDonald & Kreitman 1991) to estimate the number of contigs evolving under adaptive and neutral evolution. The McDonald–Kreitman test contrasts the number of nonsynonymous and synonymous substitutions (*D*_N_ and *D*_S_) with polymorphisms (P_N_ and P_S_). *D*_N_ and *D*_S_ for each species were obtained from the species‐specific paml analysis, where divergence was calculated from the terminal tip to the Galloanserae common ancestor, excluding the *T. guttata* outgroup. A deficit of nonsynonymous polymorphisms relative to substitutions is indicative of positive selection [(*D*_N_/*D*_S_) > (P_N_/P_S_)], and an excess of nonsynonymous polymorphisms relative to substitutions is indicative of relaxed purifying selection [(*D*_N_/*D*_S_) < (P_N_/P_S_)]. For each contig, we tested for departures from neutrality using a 2 × 2 contingency table and Pearson's chi‐squared test (Hope 1968; Patefield 1981) in r v3.1.0 (R Core Team 2014). Contigs were only included in the analysis if the sum of each marginal row and column of the 2 × 2 contingency table was greater or equal than 6 (Begun *et al*. 2007; Andolfatto 2008). We used the qvalue function in r with a false discovery rate = 0.05 and lambda = 0 to correct for multiple testing. After identifying contigs with signatures of positive selection, we tested for significant differences in the proportion of these contigs on the Z chromosome vs. the autosomes using Pearson's chi‐squared test in r.

Lastly, we used polymorphism data to test for an excess or under‐representation of Z‐linked nonsynonymous polymorphisms relative to the autosomes. Excess or underrepresentation is indicative of relaxed purifying selection or positive selection, respectively. For this analysis, we separately concatenated P_N_ and P_S_ for each species and used Pearson's chi‐squared test to test for significant differences in P_N_/P_S_ between the Z chromosome and autosomes (Mank *et al*. 2007a).

### Calculating relative effective population size of the Z chromosome

We calculated the effective population size (*N*_E_) of the Z chromosome and autosomes 1–10 for each species using two separate approaches based on π and θ.

For each contig, the number of fourfold degenerate sites (4D) and polymorphic fourfold degenerate sites (P_4D_) was calculated. Nucleotide diversity was calculated for each genomic category as π = sum P_4D_/sum 4D. Watterson's estimator of theta (θ) (Watterson 1975) was also calculated as θ = sum 4D/(sum[i = 1…*n*−1] 1/i) where *n* is the number of chromosomes in the sample. θ per site was then calculated. Finally, we recalculated π and θ using all polymorphic synonymous sites.

Effective population size was calculated separately for the Z and autosomes as *N*_E_ = (π or θ)/[4*(U*generation time)]. The mutation rate per site per year (U) was calculated separately for the Z chromosome (1.45E‐09) and autosomes (1.33E‐09) to account for male‐mutation bias, using previous Galliform estimates of Z‐linked and autosomal divergence (Dimcheff *et al*. 2002; Axelsson *et al*. 2004; van Tuinen & Dyke 2004; Mank *et al*. 2010a). U = K/2T, where K is the no of substitutions per site between homologous sequences and T is divergence time. Bootstrapping with 1000 repetitions was used to generate 95% confidence intervals for effective population size estimates.

### Tests of positive selection using gene expression

The relative role of selection vs. drift in driving Faster‐Z Evolution can be disentangled using gene expression (Baines *et al*. 2008; Mank *et al*. 2010b; Sackton *et al*. 2014). Gene expression was quantified using only adult gonad samples, because this tissue exhibits the greatest magnitude of sex‐biased transcription (Mank *et al*. 2007b; Pointer *et al*. 2013) and therefore maximizes the number of female‐biased contigs used in the analysis. Expression was estimated as reads per kilobase per million mappable reads (RPKM) and normalized to control for differences in sequencing depth across samples (Brawand *et al*. 2011).

Mean male and female RPKM of each orthogroup were calculated separately for each species, together with fold change [a measure of sex‐bias: log_2_(male RPKM)‐log_2_(female RPKM)]. A *t*‐test was used to identify significantly sex‐biased contigs, and the Benjamini–Hochberg method (FDR of 5%) (Benjamini & Hochberg 1995) used to correct for multiple testing (Mank *et al*. 2010c; Pointer *et al*. 2013; Perry *et al*. 2014). Female‐biased and male‐biased contigs were classified as significantly sex‐biased (*P* < 0.05) or sex‐limited with a log_2_ fold change of <−1 and >1, respectively. Unbiased contigs had a log_2_ fold change between <1 and >−1.

To verify that our method of defining sex bias was consistent with other approaches, we also used edger to categorize sex bias and compared the overlap between both approaches. Briefly, for each species, we extracted raw read counts for 2 RPKM filtered contigs from rsem (Li & Dewey 2011), normalized to control for differences in sequencing depth across samples using TMM in edger and tested for sex‐biased gene expression using the exactTest function in edger (Robinson & Oshlack 2010; Robinson *et al*. 2010; McCarthy *et al*. 2012). Female‐biased and male‐biased contigs were classified as above using a significant *P*‐value and log_2_ fold change of <−1 and >1, respectively. Our approach of categorizing sex bias was consistent with the results from edger, and we observe an overlap of 89–96% between expression categories as defined by both approaches.

We used three approaches to test the predictions of the selection and drift hypotheses. First, we calculated Faster‐Z for orthogroups where expression category was conserved across all six species. This was to avoid diluting significant signals of selection or drift by including orthogroups where exposure to the dominant evolutionary force has not been consistent over time due to rapid expression turnover. Mean *d*_N_, *d*_S_ and *d*_N_/*d*_S_ were calculated separately for each expression category for Z‐linked and autosomal contigs using divergence estimates from the Galloanserae analysis in codeml (Yang 2007). Bootstrapping with 1000 repetitions was used to generate 95% confidence intervals. Significant differences between genomic categories were determined using permutation tests with 1000 repetitions.

We then repeated this analysis with relaxed criteria to maximize the number of orthogroups in each expression category. Specifically, we compared the Faster‐Z Effect between putatively female‐biased contigs (defined as contigs where at least half of the species had female‐limited or significantly female‐biased expression, and the fold change was <0 across all species) and male‐biased contigs (where at least half of the species had male‐limited or significantly male‐biased expression, and the fold change was >0 across all species).

Finally, we assessed the relationship between species‐specific Faster‐Z Evolution and gene expression. For each species, we separately calculated *d*_NZ_/*d*_SZ_: *d*_NA_/*d*_SA_ for female‐, male‐ and unbiased contigs for each species as defined with *t*‐tests and fold change thresholds. Significance was assessed using permutation tests with 1000 repetitions.

### Gene ontology analysis

We used gorilla (Eden *et al*. 2007, 2009) to perform a Gene Ontology enrichment analysis to test for enriched gene function terms for Z‐linked contigs compared with the autosomes. Mouse reciprocal orthologs were identified using biomart (ensembl v.77) for Z‐linked and autosomal 1–10 orthologs. The target list contained Z‐linked orthologs and the background list contained autosomal orthologs. *P*‐values were corrected for multiple testing using the Benjamini–Hochberg method (Benjamini & Hochberg 1995).

## Results

### Faster–Z Evolution

**Figure 3:**
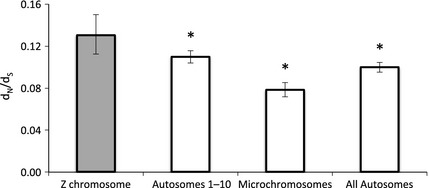
Estimates of mean *d*_N_/*d*_S_ for loci on autosomes and the Z chromosome across the Galloanserae. Synonymous and nonsynonymous divergence estimates were calculated using the branch model in paml (Galloanserae analysis). 95% confidence intervals were calculated by bootstrapping with 1000 replicates, and significant differences in *d*_N_/*d*_S_ between autosomal and Z‐linked orthogroups (permutation test, 1000 replicates) are indicated (*).

We assembled *de novo* transcriptomes for six Galloanserae species, spanning approximately 90 million years of avian evolution van Tuinen and Hedges (2001) (Fig. 2), and identified 160 Z‐linked and 2431 autosomal orthogroups. Across the Galloanserae, mean *d*_N_/*d*_S_ of the Z chromosome is significantly higher than that of the autosomes, due to significantly elevated *d*_NZ_ (Table 1, Fig. 3). There is no difference in *d*_S_ between the Z chromosomes and all autosomes (*P* = 0.865).

**Table 1:** *d*_N_, *d*_S_ and *d*_N_/*d*_S_ for Z‐linked and autosomal genes across Galloanserae clade

	Z chromosome (160 contigs)	Autosomes 1–10 (1690 contigs)	Microchromosomes (741 contigs)	All autosomes (2431 contigs)
*d*_S_ 95% CI	0.432 (0.413–0.454)	0.424 (0.417–0.432) *P* = 0.229	0.510 (0.493–0.528) *P* = 1.000	0.447 (0.440–0.454) *P* = 0.865
*d*_N_ 95% CI	0.056 (0.049–0.065)	**0.047 (0.044–0.049)** ***P*** ** = 0.007**	**0.040 (0.037–0.043)** ***P*** ** < 0.001**	**0.045 (0.042–0.047)** ***P*** ** = 0.005**

Significance values were determined from 1000 permutation tests, and bootstrapping with 1000 repetitions was used to generate 95% confidence intervals. Significant differences between autosomal and Z‐linked orthogroups are in bold.

Seven‐hundred and forty‐one autosomal orthogroups are located on microchromosomes in the chicken genome, and microchromosomes exhibit different genomic properties to the rest of the autosomes. These properties impact the nature and efficacy of selection (Burt 2002; Ellegren 2013); therefore, the fairest measure of Faster‐Z Evolution is to contrast divergence between the Z chromosome and similar‐sized autosomes 1–10 (Mank *et al*. 2010b). We identified 1690 orthogroups located on autosomes 1–10. Mean *d*_NZ_/*d*_SZ_ and *d*_NZ_ are both significantly higher than mean *d*_N_/*d*_S_ and *d*_N_ of autosomal 1–10 orthogroups (Table 1, Fig. 3). This pattern is consistent with the results of the previous analysis using all autosomes, and with previous estimates of Faster‐Z Evolution in birds (Mank *et al*. 2007a, 2010b; Dalloul *et al*. 2010; Ellegren *et al*. 2012; Wang *et al*. 2014a). For the rest of the manuscript, autosomal will refer to autosomal 1–10 orthogroups and Faster‐Z will refer to the comparison between Z‐linked and autosomal 1–10 orthogroups *d*_NZ_/*d*_SZ_: *d*_NA_/*d*_SA_.

In each of the six Galloanserae species, *d*_NZ_/*d*_SZ_ is higher than *d*_NA_/*d*_SA_ based on the species‐specific analysis, and there is interspecific variation in the magnitude of this difference (Table 2). We find no significant difference in *d*_S_ between the Z chromosome and autosomes for any species, consistent with previous findings that male‐biased mutation rate is weak across the Galloanserae (Bartosch‐Harlid *et al*. 2003; Axelsson *et al*. 2004). This suggests that Z‐linked mutation rate does not vary significantly across the six species (addressed further in the Discussion).

**Table 2:** *d*_N_, *d*_S_ and *d*_N_/*d*_S_ for Z‐linked and autosomal genes across Galloanserae species

Species	Z chromosome			Autosomes 1–10			Faster‐Z Effect
	*d*_N_ (95% CI)	*d*_S_ (95% CI)	*d*_N_/*d*_S_ (95% CI)	*d*_N_ (95% CI)	*d*_S_ (95% CI)	*d*_N_/*d*_S_ (95% CI)	*d*_NZ_/*d*_SZ_: *d*_NA_/*d*_SA_ (95% CI)
*Meleagris gallopavo*	0.023 (0.020–0.027)	0.163 (0.155–0.170)	0.144 (0.123–0.165)	**0.019 (0.018**–**0.020)** ***P*** ** = 0.005**	0.158 (0.154–0.161) *P* = 0.168	**0.120 (0.113**–**0.127)** ***P*** ** = 0.011**	1.205 (1.035–1.390)
*Phasianus colchicus*	0.021 (0.018–0.025)	0.161 (0.153–0.168)	0.134 (0.114–0.154)	**0.018 (0.017**–**0.020)** ***P*** ** = 0.035**	0.157 (0.153–0.160) *P* = 0.215	0.118 (0.111–0.125) *P* = 0.061	1.137 (0.961–1.331)
*Numida meleagris*	0.019 (0.016–0.022)	0.133 (0.127–0.140)	0.140 (0.119–0.162)	**0.016 (0.015**–**0.017)** ***P*** ** = 0.041**	0.132 (0.129–0.135) *P* = 0.393	**0.123 (0.116**–**0.130)** ***P*** ** = 0.049**	1.140 (0.965–1.332)
*Anas platyrhynchos*	0.015 (0.012–0.018)	0.116 (0.108–0.126)	0.131 (0.107–0.155)	**0.013 (0.012**–**0.014)** ***P*** ** = 0.030**	0.116 (0.113–0.119) *P* = 0.518	**0.109 (0.103**–**0.116)** ***P*** ** = 0.024**	1.200 (0.974–1.457)
*Anser cygnoides*	0.012 (0.010–0.015)	0.100 (0.093–0.107)	0.125 (0.103–0.148)	0.011 (0.010–0.012) *P* = 0.083	0.099 (0.097–0.101) *P* = 0.378	0.111 (0.105–0.118) *P* = 0.083	1.129 (0.939–1.360)
*Pavo cristatus*	0.020 (0.017–0.023)	0.147 (0.139–0.154)	0.134 (0.114–0.157)	0.017 (0.016–0.018) *P* = 0.068	0.147 (0.144–0.150) *P* = 0.502	0.118 (0.112–0.125) *P* = 0.056	1.133 (0.951–1.303)

Significance values were determined from 1000 permutation tests and bootstrapping with 1000 repetitions was used to generate 95% confidence intervals. Significant differences between autosomal and Z‐linked orthologs are shown in bold.

### Variation in sperm competition drives Faster‐Z Evolution

**Figure 4:**
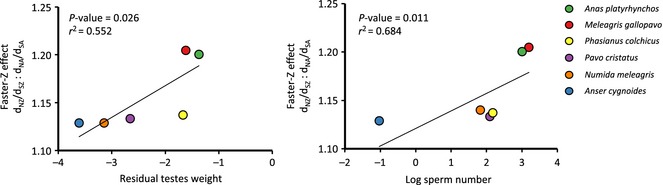
Phylogenetically controlled regression between proxies of sperm competition and Faster‐Z Effect. Data points are raw species values but *P*‐values and *r* ^2^ estimates were calculated using phylogenetic generalized least squares regression with maximum likelihood and 1000 runs for each analysis. Autosomes refers to macrochromosomes (autosomes 1–10).

The intensity of sperm competition, a widely used indicator of postcopulatory sexual selection and therefore one measure of variance in male mating success, is strongly predicted by relative testes weight and sperm number in birds (Moller 1991; Birkhead & Moller 1998; Pitcher *et al*. 2005). We recovered a significant positive association between magnitude of Faster‐Z Evolution and both log sperm number (*r* ^2^ = 0.684, *P* = 0.011, *t*_4_ = 3.629) and residual testes weight (*r* ^2^ = 0.552, *P* = 0.026, *t*_4_ = 2.744) after correcting for phylogeny (Fig. 4). To test the strength of these associations, we sequentially removed each species and repeated the analyses (Table S5). Despite the reduction in sample size and therefore statistical power, there was no change to the significance or direction of the slope for log sperm number. For residual testes weight, there was no change to the direction of the slope but when either *A. cygnoides* or *A. platyrhynchos* was excluded, the relationship was nonsignificant (Table S5).

There are two plausible explanations for our finding that the magnitude of Z‐linked divergence increases with increasing female promiscuity. A recent study in silk moths has shown that Faster‐Z Evolution is adaptive, and results from increased efficacy of selection acting on recessive advantageous mutations in the hemizygous sex (Sackton *et al*. 2014). Conversely, a study in birds suggested that avian Faster‐Z Evolution is a neutral process, driven by relaxed efficacy of purifying selection as a consequence of relative differences in *N*_EZ_/*N*_EA_ (Mank *et al*. 2010b). Under the latter hypothesis, variation in male reproductive success, associated with sexual selection, is predicted to alter the relationship between *N*_EZ_ and *N*_EA_, and therefore the relative magnitude of drift acting on the Z chromosome (Charlesworth *et al*. 1993; Vicoso & Charlesworth 2009). Specifically, with increasing variance in male reproductive success, relative *N*_EZ_ decreases, resulting in greater magnitude of drift and therefore Faster‐Z Effect (Wright & Mank 2013).

We use sequence divergence, polymorphism and expression data to test whether the relationship between female promiscuity and Faster‐Z Evolution is adaptive or neutral.

### Estimates of relative *N*_EZ_

After filtering for quality and read depth, across Z‐linked and autosomal 1–10 contigs, we identified 12 436 SNPs in *A. platyrhynchos,* 4584 in *M. gallopavo,* 6850 in *P. colchicus,* 5205 in *N. meleagris,* 2012 in *P. cristatus* and 8128 in *A. cygnoides* (Table S3).

For each species, we calculated the effective population size of the Z chromosome (*N*_EZ_) and autosomes 1–10 (*N*_EA_) using a number of approaches. We accounted for male‐biased mutation rate and generation time using previous Galliform estimates (Dimcheff *et al*. 2002; Axelsson *et al*. 2004; van Tuinen & Dyke 2004; Mank *et al*. 2010a) (Table 3, Tables S6, S7 and S8, Supporting Information) (Vicoso & Charlesworth 2009). Under strict monogamy, *N*_EZ_ is predicted to equal ¾ *N*_EA_. For all species with the exception of *P. cristatus*,* N*_EZ_ was significantly <¾ *N*_EA_. However, the 95% CI for this species was unusually wide, probably as a result of the low frequency of SNPs detected (Table S3).

**Table 3:** Effective population size estimates of the Z chromosome and autosomes

Species	*N*_EZ_ (E + 05) (95% CI)	*N*_EA1–10_ (E + 05) (95% CI)	*N*_EZ_/*N*_EA1–10_ (95% CI)
*Meleagris gallopavo*	1.761 (1.087–2.702)	6.047 (5.656–6.469)	0.291 (0.179–0.426)
*Phasianus colchicus*	3.188 (2.308–4.210)	9.481 (8.948–10.054)	0.336 (0.234–0.460)
*Numida meleagris*	1.695 (0.773–3.213)	7.233 (6.682–7.848)	0.234 (0.103–0.423)
*Anas platyrhynchos*	6.150 (3.927–8.758)	18.427 (17.447–19.544)	0.334 (0.209–0.470)
*Anser cygnoides*	4.045 (2.774–5.591)	10.894 (10.233–11.570)	0.371 (0.250–0.529)
*Pavo cristatus*	1.088 (0.167–2.811)	2.393 (2.095–2.697)	0.455 (0.057–1.227)

*N*_E_ was calculated using the same method as Mank *et al*. 2010b;. Mutation rate estimates are from Axelsson *et al*. 2004; Dimcheff *et al*. 2002 and van Tuinen & Dyke 2004.

Minor allele frequency threshold of 0.15.

Nucleotide diversity (π) was calculating using fourfold degenerate sites.

The relationship between *N*_EZ_/*N*_EA_ and sperm number, residual testes weight or Faster‐Z was not statistically significant (sperm number: *r* ^2^ = 0.083, *P* = 0.252, *t*_4_ = 0.735; residual testes weight: *r* ^2^ = 0.068, *P* = 0.275, *t*_4_ = 0.656; Faster‐Z: *r* ^2^ = 0.220, *P* = 0.132, *t*_4_ = 1.300; Table S9, Supporting Information). Additionally, the autosomal effective population size of *P. cristatus* is significantly smaller than the other six species, indicating either a very recent bottleneck or variation in family structure across the individuals sampled in this study. This finding hints at the sensitivity of *N*_E_ calculations to many factors (Hartl & Clark 2007), including recombination rate and recent demographic perturbations (Pool & Nielsen 2007). This may explain both the unusually low *N*_E_ estimates in *P. cristatus* as well as the lack of significant association between *N*_EZ_/*N*_EA_ and measures of sperm competition (addressed further in the Discussion).

### Tests of positive selection

We used sequence and polymorphism data from our six species to test whether selection is more effective for Z‐linked vs. autosomal loci. Using the site‐model test in codeml, we found significant evidence for positive selection acting on 5/160 Z‐linked loci (1/160 after sequential Bonferroni's correction) and 51/1690 autosomal loci (5/1690 after sequential Bonferroni's correction) (Table 4, Table S10, Supporting Information). There was no significant difference in the proportion of positively selected loci on the Z chromosome or autosomes 1–10 either before or after multiple testing correction (χ^2^, d.f. = 1, *P* > 0.400 in both comparisons). This indicates that selection is not more effective on the Z chromosome; however, the power of this analysis is limited by the low number of total contigs under positive selection.

**Table 4:** Site‐model test results for contigs under positive selection

*G. gallus* ortholog^a^	Chromosome	ω	Proportion of sites	M1a likelihood ratio	M2a likelihood ratio	LRT	*P*‐value	*P*‐fdr value^b^
22552	1	2.897	0.122	−6535.857	−6522.227	27.259	<0.001	0.003
21101	1	4.155	0.033	−14063.297	−14050.286	26.023	<0.001	0.006
31776	3	4.608	0.130	−1270.098	−1256.430	27.337	<0.001	0.003
39919	6	4.226	0.310	−1630.735	−1611.278	38.915	<0.001	<0.001
03831	8	4.817	0.080	−9607.226	−9560.287	93.878	<0.001	<0.001
10504	15	3.343	0.072	−5389.616	−5375.473	28.287	<0.001	0.002
01868	20	9.422	0.013	−4192.958	−4179.195	27.526	<0.001	0.003
02022	28	4.914	0.068	−2768.690	−2753.634	30.110	<0.001	0.001

a ENSGALT000000.

b Sequential Bonferroni's correction (Holm 1979).

We next used polymorphism data to test for deviations from neutrality. With the exception of *N. meleagris* and *P. cristatus*, p_NZ_/p_SZ_ is significantly greater than p_NA_/p_SA_ (Table 5, Table S11, Supporting Information). This finding of excess nonsynonymous polymorphism on the Z chromosome relative to the autosomes suggests that selection is less effective at removing mildly deleterious mutations from the Z chromosome. This finding is consistent with the drift hypothesis of Faster‐Z, rather than the adaptive hypothesis. Interestingly, *N. meleagris* Z chromosome exhibits a nonsignificant deficit of p_N_, potentially as a consequence of monogamy, which would maximize *N*_EZ_/*N*_EA_ and therefore the potential of selection to act on the Z chromosome in this species.

**Table 5:** p_N_, p_S_ and p_N_/p_S_ for Z‐linked and autosomal genes across Galloanserae species

Species	Z chromosome			Autosomes 1–10			Faster‐Z Effect
	p_N_ (95% CI)	p_S_ (95% CI)	p_N_/p_S_ (95% CI)	p_N_ (95% CI)	p_S_ (95% CI)	p_N_/p_S_ (95% CI)	p_NZ_/p_SZ_: p_NA_/p_SA_ (95% CI)
*Meleagris gallopavo*	0.000 (0.000–0.000)	0.001 (0.001–0.002)	0.176 (0.112–0.256)	0.000 (0.000–0.001) *P* = 1.000	0.005 (0.005–0.005) *P* = 1.000	**0.102 (0.093–0.111)** ***P*** ** < 0.001**	1.721 (1.039–2.716)
*Phasianus colchicus*	0.000 (0.000–0.001)	0.002 (0.002–0.003)	0.162 (0.109–0.236)	0.001 (0.001–0.001) *P* = 1.000	0.007 (0.007–0.008) *P* = 1.000	**0.095 (0.088–0.103)** ***P*** ** < 0.001**	1.704 (1.122–2.546)
*Numida meleagris*	0.000 (0.000–0.000)	0.002 (0.001–0.003)	0.083 (0.050–0.150)	0.001 (0.001–0.001) *P* = 1.000	0.005 (0.005–0.006) *P* = 1.000	0.102 (0.093–0.112) *P* = 0.897	0.813 (0.483–1.370)
*Anas platyrhynchos*	0.001 (0.000–0.001)	0.005 (0.004–0.008)	0.103 (0.064–0.156)	0.001 (0.001–0.001) *P* = 1.000	0.014 (0.013–0.015) *P* = 1.000	**0.072 (0.066–0.078)** ***P*** ** = 0.002**	1.426 (0.863–2.120)
*Anser cygnoides*	0.001 (0.000–0.001)	0.003 (0.002–0.004)	0.177 (0.109–0.262)	0.001 (0.001–0.001) *P* = 0.999	0.008 (0.008–0.009) *P* = 1.000	**0.108 (0.099–0.117)** ***P*** ** < 0.001**	1.642 (1.009–2.427)
*Pavo cristatus*	0.000 (0.000–0.000)	0.001 (0.000–0.002)	0.173 (0.096–0.541)	0.000 (0.000–0.000) *P* = 0.930	0.002 (0.002–0.002) *P* = 1.000	0.134 (0.116–0.156**)** *P* = 0.137	1.293 (0.681–4.002)

Significance values were determined from 1000 permutation tests, and bootstrapping with 1000 repetitions was used to generate 95% confidence intervals.

Significant differences between autosomal and Z‐linked orthologs are shown in bold.

Minor allele frequency threshold of 0.15.

For each species, we estimated the number of contigs evolving under adaptive evolution using the McDonald–Kreitman test (McDonald & Kreitman 1991). This test contrasts the number of nonsynonymous and synonymous substitutions (*D*_N_ and *D*_S_) with polymorphisms (P_N_ and P_S_) for each contig. An excess of nonsynonymous substitutions relative to polymorphism is indicative of positive selection [(*D*_N_/*D*_S_) > (P_N_/P_S_)], and under‐representation of nonsynonymous substitutions relative to polymorphism is indicative of relaxed purifying selection [(*D*_N_/*D*_S_) < (P_N_/P_S_)]. We detected no Z‐linked contigs with signatures of positive selection, and there was no difference between the Z chromosome and autosomes 1–10 in the proportion of loci under positive selection in any species (χ^2^, d.f. = 1, *P* > 0.500 in all cases) (Table S12, Supporting Information). However, only contigs with sufficient numbers of substitutions and polymorphisms were included in the analysis (Begun *et al*. 2007; Andolfatto 2008), and therefore, our ability to draw species‐specific conclusions is limited by low sample sizes.

Lastly, for each species, we concatenated the number of P_N_ and P_S_ across all Z‐linked and all autosomal 1–10 contigs separately (Table 6, Table S13, Supporting Information) and tested for significant differences between Z‐linked and autosomal P_N_/P_S_. For each species, there is a significant excess of Z‐linked nonsynonymous polymorphism relative to the autosomes for all species with the exceptions of *P. cristatus* and *N. meleagris*. This is again consistent with a reduction in the power of selection to remove mildly deleterious alleles from this chromosome.

**Table 6:** Significant differences between nonsynonymous and synonymous polymorphism on the Z chromosome and autosomes

Species	Z chromosome		Autosomes 1–10		Faster‐Z Effect
	P_N_	P_S_	P_N_	P_S_	P_NZ/_P_SZ_: P_NA/_P_SA_ *P*‐value
*Meleagris gallopavo*	51	83	1174	3276	**1.715** ***P*** ** = 0.004**
*Phasianus colchicus*	89	157	1654	4950	**1.700** ***P*** ** < 0.001**
*Numida meleagris*	29	100	1339	3737	0.809 *P* = 0.372
*Anas platyrhynchos*	126	351	2417	9542	**1.417** ***P*** ** = 0.001**
*Anser cygnoides*	127	206	2138	5657	**1.631** ***P*** ** < 0.001**
*Pavo cristatus*	38	63	610	1301	1.286 *P* = 0.277

Significant differences were determined using Pearson's chi‐squared test in R.

Significant differences between autosomal and Z‐linked orthologs are shown in bold.

Minor allele frequency threshold of 0.15.

The lack of difference in Z‐linked and autosomal nonsynonymous polymorphism in *P. cristatus* and *N. meleagris* could be attributed to a number of factors. It could reflect biological differences in sexual selection and therefore the magnitude of drift acting on the Z chromosome. However, although this explanation is consistent with the monogamous mating system of *N. meleagris*, it is not consistent with the *P. cristatus,* which exhibits a lek mating system (Petrie *et al*. 1999). More likely, this pattern reflects the limitations of polymorphism data and the difficulty in controlling for family structure and demographic effects (Hartl & Clark 2007). For example, the number of SNPs in *P. cristatus* is much lower than the other five species, and therefore, the statistical power of this analysis is limited (Table 6).

Differences in gene content between the sex chromosomes and autosomes can contribute to observed patterns of Faster‐Z/X (Meisel & Connallon 2013) by biasing the potential for positive selection in different genomic categories. However, the results of our gorilla functional enrichment test reveal no significantly enriched gene ontology terms for Z‐linked orthogroups compared with autosomes 1–10 after correcting for multiple tests.

### Gene expression

We used gene expression data from gonads of our six avian species to identify the dominant force driving Faster‐Z Evolution across the Galloanserae clade. If Faster‐Z Evolution is adaptive and driven by increased efficacy of selection acting on recessive mutations in the hemizygous sex, we predict the Faster‐Z Effect to be largest for female‐biased, followed by unbiased and then male‐biased genes. If it is due to neutral causes, there will be no difference in the rate of Faster‐Z Evolution among expression classes (Baines *et al*. 2008; Mank *et al*. 2010b; Sackton *et al*. 2014). We tested this prediction at three levels in our data.

**Figure 5:**
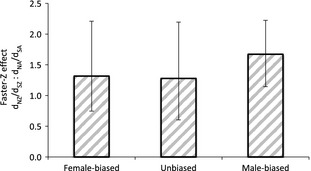
Estimates of mean Faster‐Z across sex‐biased gene expression categories. Sex bias was defined using fold change thresholds and *t*‐tests. 95% confidence intervals were calculated by bootstrapping with 1000 replicates. Autosomal orthologs were limited to chromosomes 1–10.

First, we identified orthogroups with consistent male‐, female‐ and unbiased expression across all six species, thereby excluding any orthogroups where the nature of sex‐bias, and therefore exposure to the dominant evolutionary force, has varied over Galloanserae evolutionary history. The rapid change in sex bias across this clade (Harrison *et al*. in press) means that relatively few genes are consistently sex‐biased in our data set, resulting in 17 male‐biased, 9 female‐biased and 7 unbiased Z‐linked orthogroups alongside 104 male‐biased, 116 female‐biased and 205 unbiased autosomal orthogroups. Among these gene sets, there was no significant difference in Faster‐Z Effect (male‐biased vs. female‐biased *P* = 0.542, female‐biased vs. unbiased *P* = 1.000, male‐biased vs. unbiased *P* = 0.616, all two‐tailed pairwise permutation tests with 1000 repetitions), shown in Fig. 5.

To exclude the possibility that we lack statistical power to distinguish between drift and selection due to low sample sizes, we next repeated the analysis and relaxed the definition of sex bias (see Materials and methods). In doing so, we nearly doubled the number of orthogroups in each expression category; identifying 54 male‐biased and 15 female‐biased Z‐linked orthogroups, together with 347 male‐biased and 319 female‐biased autosomal orthogroups. Again, there was no significant difference in Faster‐Z Effect between these gene sets (*P* = 0.916, permutation test, 1000 repetitions), with female‐biased *d*_NZ_/*d*_SZ_: *d*_NA_/*d*_SA_ = 1.491 (95% CI = 0.997−2.137) and male‐biased *d*_NZ_/*d*_SZ_: *d*_NA_/*d*_SA_ = 1.456 (95% CI = 1.112−1.869).

Finally, we assessed whether there was any species‐specific pattern in Faster‐Z Evolution across male‐, female‐ and unbiased contigs. There is no significant difference between Faster‐Z of any expression category in any species after correction for multiple testing, with the exception of *N. meleagris* where we found a significantly larger Faster‐Z Effect for male‐biased compared with unbiased contigs (Tables S14 and S15, Supporting Information). At all three levels of analysis, our expression data are consistent with Faster‐Z Evolution resulting predominantly from neutral forces.

## Discussion

Faster rates of coding sequence evolution on the Z chromosome relative to the autosomes have been observed across a wide range of species (Mank *et al*. 2007a, 2010b; Dalloul *et al*. 2010; Ellegren *et al*. 2012; Sackton *et al*. 2014; Wang *et al*. 2014a,b); however, the underlying cause is unclear. Indirect evidence from an expression‐based approach suggests that avian Faster‐Z Evolution is driven by genetic drift (Mank *et al*. 2010b), but a recent study in silk moths postulated an adaptive explanation (Sackton *et al*. 2014). To determine the cause of Faster‐Z Evolution in birds, we assembled *de novo* transcriptomes for six Galloanserae species, spanning 90 million years of avian evolution and combined expression, sequence and polymorphism data with measures of sperm competition and promiscuity. We present the first empirical evidence demonstrating the positive relationship between the Faster‐Z Effect and measures of postcopulatory sexual selection and variance in male reproductive success.

This pattern is consistent with a considerable body of theory predicting that Faster‐Z Evolution in birds is driven by changes in the relative strength of genetic drift as a result of increased variance in male reproductive success (Vicoso & Charlesworth 2009). In support of the predominant role of genetic drift in shaping rates of Z chromosome evolution, we used multiple sequence‐, polymorphism‐ and expression‐based approaches. Our expression analysis is consistent with previous work that found no difference in Faster‐Z Evolution among sex‐biased expression categories (Mank *et al*. 2010b). However, our analysis significantly extends this previous work by incorporating tests of positive selection based on divergence and polymorphism. The results from these multiple lines of evidence are broadly convergent, indicating that selection is not more effective on the Z chromosome. We conclude that Faster‐Z Evolution in birds is due primarily to relaxed power of purifying selection and that the magnitude of this effect is dependent on the nature of sexual selection.

### Promiscuity and sperm competition are drivers of Faster‐Z Evolution

Changes in the skew of male reproductive success are commonly associated with promiscuity and the intensity of postcopulatory sexual selection (Andersson 1994), both of which decrease the *N*_EZ_/*N*_EA_ ratio. If Faster‐Z is neutral and nonadaptive, we predict that the magnitude of Faster‐Z Evolution should increase as *N*_EZ_/*N*_EA_ decreases (Vicoso & Charlesworth 2009), and therefore, we should expect both lower *N*_EZ_/*N*_EA_ and increased rates of Faster‐Z Evolution in promiscuous compared with monogamous populations (Fig. 1).

We uncovered a significant and positive association between the magnitude of Faster‐Z and relative testes weight and sperm number, both reliable predictors of the intensity of sperm competition in birds (Fig. 4) (Moller 1991; Birkhead & Moller 1998; Pitcher *et al*. 2005). Sperm competition is a widely used indicator of the strength of postcopulatory sexual selection and therefore a good proxy for variance in male mating success and the magnitude of drift acting on the Z chromosome (Moller 1991; Birkhead & Moller 1998; Dorus *et al*. 2004). It is even possible we have underestimated the role of male mating success in driving Z chromosome divergence, as the birds sampled in this study have a lower testes weight than expected given their body weight (Pitcher *et al*. 2005).

Although the relationship between *N*_EZ_/*N*_EA_ and sperm number or residual testes weight was not significant, *N*_EZ_/*N*_EA_ across the Galloanserae is consistent with the nonadaptive hypothesis of Faster‐Z Evolution (Vicoso & Charlesworth 2009) and is significantly less than the 0.75 predicted under strict monogamy, with the exception of *P. cristatus* (Table 3). We calculated effective population size using parameters estimated from previous Galliform studies (Dimcheff *et al*. 2002; Axelsson *et al*. 2004; van Tuinen & Dyke 2004; Mank *et al*. 2010a), and although mutation rate, male‐biased mutation and generation time are not expected to vary substantially across the Galloanserae, we might expect slight differences. Overall *N*_E_ is also predicted to have a large effect on the magnitude of Faster‐Z and relative contribution of nonadaptive and adaptive evolutionary forces. However, patterns of autosomal *N*_E_ do not reflect differences in Faster‐Z across species.

Polymorphism estimates are sensitive to recent demographic perturbations, bottlenecks and recombination rate (Hartl & Clark 2007). Changes in population size have been shown to differentially impact *N*_EZ_ relative to *N*_EA_ and variation in population history across the Galloanserae may contribute to the lack of a significant relationship between *N*_EZ_/*N*_EA_ and measures of promiscuity and sperm competition (Pool & Nielsen 2007). Previous attempts to estimate *N*_EZ_/*N*_EA_ in birds (Corl & Ellegren 2012) showed sizable variation from what would be predicted by mating system, suggesting that *N*_EZ_/*N*_EA_ estimates may simply be too inaccurate for the types of analyses used here. Because divergence data are not as sensitive to recent demographic perturbations, it can be argued that it is a fairer test for the role of male mating success and sperm competition in Faster‐Z Evolution.

### Tests of positive selection

We used sequence and polymorphism data to test the relative strength of selection on the Z chromosome vs. autosomes. In both the site‐model tests in paml as well as species‐specific McDonald–Kreitman tests, there was no difference in the proportion of positively selected loci on the Z chromosome compared with the autosomes. The McDonald–Kreitman test is limited to sequences with sufficient numbers of substitutions and polymorphisms (McDonald & Kreitman 1991; Andolfatto 2008), and this restricted our analysis to a handful of Z‐linked contigs. Therefore, to maximize the power of our data set, we concatenated polymorphism data across all Z‐linked and autosomal contigs (Mank *et al*. 2007a). For the majority of species, an excess of Z‐linked nonsynonymous polymorphism relative to the autosomes was observed, suggesting that selection is less able to purge mildly deleterious alleles from the Z chromosome. This pattern is consistent with the theoretical expectations of elevated levels of genetic drift. We would expect the opposite pattern, a deficit of Z‐linked nonsynonymous polymorphism, under both positive and purifying selection.

Differences in gene content between the sex chromosomes and autosomes can bias the potential for positive selection to act on different genomic categories, and therefore may contribute to our observed patterns of Faster‐Z (Meisel & Connallon 2013). The avian Z chromosome is enriched in male‐biased genes (Mank & Ellegren 2009), which typically exhibit rapid rates of evolution (Meisel 2011; Parsch & Ellegren 2013). However, we do not find an elevated Faster‐Z Effect for male‐biased genes, and the results of our gorilla functional enrichment analysis reinforce that differences in gene content are not likely to drive the pattern of Faster‐Z we observe.

Overall, we failed to detect any indication that selection is more effective for Z‐linked loci, consistent with the nonadaptive explanations for Faster‐Z Evolution. However, it is important to note that our analyses are limited to orthologs conserved across 90 million years, and conservation across this span of time suggests that purifying selection is a dominant force acting on these genes. The important role of purifying selection in this gene set may bias our ability to detect positive selection using this data set. Nevertheless, our neutral explanation of Faster‐Z is consistent with previous work indicating that sex chromosome dosage compensation status mediates the contribution of positive selection to Faster‐Z Effect (Charlesworth *et al*. 1987; Mank 2009). Theory predicts that the adaptive component of Faster‐Z is weaker in species with incomplete dosage compensation, such as birds (Ellegren *et al*. 2007; Mank 2009; Itoh *et al*. 2010; Uebbing *et al*. 2013), compared to those with complete dosage compensation.

Theory predicts that the magnitude of Faster‐Z Effect should increase as *N*_EZ_/*N*_EA_ decreases (Vicoso & Charlesworth 2009), and therefore, we should expect increased rates of Faster‐Z Evolution in promiscuous compared with monogamous populations. This prediction is consistent with our finding that Faster‐Z is positively correlated with the intensity of sperm competition, and therefore variance in male reproductive success.

### Faster‐Z vs. Faster‐X Evolution

Faster rates of coding sequence divergence have repeatedly been documented on the X and Z chromosomes relative to the autosomes, and there is considerable variation in the magnitude of this difference across species (Meisel & Connallon 2013). Moreover, there is a stark contrast between our results and those of Faster‐X Evolution in *Drosophila* and mammals, where X‐linked male‐biased genes evolve more rapidly than unbiased and female‐biased genes (Khaitovich *et al*. 2005; Baines *et al*. 2008; Grath & Parsch 2012). This pattern is consistent with an adaptive explanation of Faster‐X Evolution driven by increased efficacy of selection acting on recessive mutations in the heterogametic sex. In addition, there is considerable evidence for signatures of adaptation on the X chromosome across many species (Thornton & Long 2005; Baines *et al*. 2008; Hvilsom *et al*. 2012; Langley *et al*. 2012).

The empirical evidence for neutral vs. adaptive explanations of Faster‐Z and Faster‐X Evolution, respectively, is supported by theoretical predictions (Vicoso & Charlesworth 2009). As variance in male reproductive fitness increases, *N*_EZ_ < ¾ *N*_EA_, reducing the ability of selection to purge mildly deleterious alleles. In contrast, *N*_EX_ > ¾ *N*_EA_ under increased variance in male reproductive success, indicating that Faster‐X is more often due to positive selection acting on recessive mutations exposed in the heterogametic sex. However, a recent study in silk moths (Sackton *et al*. 2014) indicates that this prediction may not hold for all female heterogametic species and is dependent on numerous other factors, including overall population size and sex‐specific recombination rates (Connallon *et al*. 2012).

### Male‐biased mutation

The relative rate of Z‐linked divergence is thought to be influenced by multiple factors, not only variance in male reproductive success (Kirkpatrick & Hall 2004a; Connallon *et al*. 2012). The number of cell divisions, and therefore potential for mutations, is inherently higher in spermatogenesis compared with oogenesis. This male‐biased mutation has been documented across a number of species (Bartosch‐Harlid *et al*. 2003; Axelsson *et al*. 2004; Xu *et al*. 2012), and as the Z chromosome is present more often in males than females, it could contribute to the observed differences in relative Z‐linked divergence (Kirkpatrick & Hall 2004a; Xu *et al*. 2012). However, previous estimates indicate the magnitude of male‐biased mutation may be relatively weak across the Galloanserae (Bartosch‐Harlid *et al*. 2003), ranging from 1.6 to 3.8 in Anseriformes (Wang *et al*. 2014b) and 1.7 to 2.52 in Galliformes (Axelsson *et al*. 2004). We failed to find a significant difference between *d*_SZ_ and *d*_SA_ in any species indicating that male‐mutation bias does not vary significantly across this clade. This is consistent with the observation that the wild species in this study are seasonal breeders where spermatogenesis ceases in the nonbreeding season. Consequentially, the difference in number of meiotic cell divisions between males and females is reduced, and therefore, the potential for male‐biased mutation is lower. In contrast, many previous estimates of male‐biased mutation were based on domesticated species with continuous breeding cycles and spermatogenesis (Bartosch‐Harlid *et al*. 2003; Axelsson *et al*. 2004). However, it is possible there is also a confounding effect of Z‐linked codon usage bias, an excess of which has been observed on the *Drosophila* X chromosome (Singh *et al*. 2008).

### Sexual selection and the Z chromosome

The sex chromosomes are predicted to play a disproportionate role in encoding sex‐specific fitness due to their unequal inheritance pattern (Rice 1984). The Z chromosome in particular is thought to foster tight linkage between female preference genes and flashy male traits, and promote rapid evolution of some types of sexually selected traits (Rice 1984; Reeve & Pfennig 2003; Kirkpatrick & Hall 2004b). However, evidence that the Z chromosome harbours genes encoding sexually dimorphic phenotypes is mixed (Dean & Mank 2014). Z‐linked male plumage genes have been documented in flycatchers (Saetre *et al*. 2003; Saether *et al*. 2007), but other studies have failed to find an association between sexually dimorphic traits and sex linkage (Knief *et al*. 2012; Schielzeth *et al*. 2012; Pointer *et al*. 2013). Our findings may help explain this discrepancy between theoretical and empirical data. The low effective population size of the Z chromosome relative to the autosomes may weaken the efficacy of sex‐specific selection, particularly in the species under the strongest sexual selection regimes. This may limit the adaptive role of the Z chromosome in general, and in particular its role in encoding sexually selected traits. Given this, it is important to note that our results do not exclude the potential for selection acting on the Z chromosome, but suggests that relaxed purifying selection is more dominant on the Z chromosome relative to the autosomes.

## Conclusions

We assessed the magnitude and drivers of Faster‐Z Evolution across a clade of birds spanning 90 million years of evolution. Our analysis combines expression, sequence and polymorphism data with measures of sperm competition and promiscuity. The results from these multiple lines of evidence are broadly convergent, indicating that selection is less effective on the Z chromosome, and suggesting that Faster‐Z Evolution in birds is due primarily to genetic drift. Moreover, we present the first empirical evidence demonstrating the positive relationship between the Faster‐Z Effect and measures of promiscuity and sperm competition, and therefore variance in male mating success.

A.E.W., M.A.P. and J.E.M. were involved in data collection. A.E.W. and J.E.M. designed the research. A.E.W., P.W.H., F.Z., S.H.M. and J.E.M. analysed the data and wrote the paper.

## Data accessibility

Illumina raw reads: SRA: PRJNA271731.

Trinity assembly, RPKM data, sequence alignments and SNP data: Dryad: doi:10.5061/dryad.4gv50.

## Supporting information


**Table S1** Assembly statistics for each Galloanserae species.
**Table S2** Assembly statistics for each sample.
**Table S3** Number of nonsynonymous and synonymous polymorphic and fixed sites at a minor allele frequency threshold of 0.15.
**Table S4** Number of nonsynonymous and synonymous polymorphic and fixed sites at a minor allele frequency threshold of 0.25.
**Table S5** Phylogenetically controlled regression analyses between Faster‐Z and log sperm number, and residual testes weight.
**Table S6** Nucleotide diversity estimates of the Z chromosome and autosomes.
**Table S7**. Effective population size estimates of the Z chromosome and autosomes.
**Table S8** Effective population size estimates of the Z chromosome and autosomes calculated using Watterson's estimation of theta.
**Table S9** Phylogenetically controlled regression analyses between different measures of NEZ/NEA and residual testes weight, log sperm number, and Faster‐Z.
**Table S10** Site model test results for contigs with signatures of positive selection.
**Table S11** pN, Ps and pN/pS for Z‐linked and autosomal genes across Galloanserae species with a minor allele frequency of 0.25.
**Table S12** McDonald Kreitman test results.
**Table S13** Significant differences between nonsynonymous and synonymous polymorphism on the Z chromosome and autosomes with a minor allele frequency threshold of 0.25.
**Table S14** Faster‐Z Effect across expression classes.
**Table S15** Differences in Faster‐Z Effect between expression classes.
