# Supplementary material for: Variation in promiscuity and sexual selection drives avian rate of Faster‐Z evolution
Source: Mol Ecol. 2015 Mar 16;24(6):1218–35. doi: 10.1111/mec.13113 (PMC4737241; doi:10.1111/mec.13113)
Supplement: Supplementary file 1 — Table S1 Assembly statistics for each Galloanserae species. Table S2 Assembly statistics for each sample. Table S3 Number of nonsynonymous and synonymous polymorphic and fixed sites at a minor allele frequency threshold of 0.15. Table S4 Number of nonsynonymous and synonymous polymorphic and fixed sites at a minor allele frequency threshold of 0.25. Table S5 Phylogenetically controlled regression analyses between Faster‐Z and log sperm number, and residual testes weight. Table S6 Nucleotide diversity estimates of the Z chromosome and autosomes. Table S7. Effective population size estimates of the Z chromosome and autosomes. Table S8 Effective population size estimates of the Z chromosome and autosomes calculated using Watterson's estimation of theta. Table S9 Phylogenetically controlled regression analyses between different measures of NEZ/NEA and residual testes weight, log sperm number, and Faster‐Z. Table S10 Site model test results for contigs with signatures of positive selection. Table S11 pN, Ps and pN/pS for Z‐linked and autosomal genes across Galloanserae species with a minor allele frequency of 0.25. Table S12 McDonald Kreitman test results. Table S13 Significant differences between nonsynonymous and synonymous polymorphism on the Z chromosome and autosomes with a minor allele frequency threshold of 0.25. Table S14 Faster‐Z Effect across expression classes. Table S15 Differences in Faster‐Z Effect between expression classes. [file MEC-24-1218-s001.pdf]

Supplementary Table 1. Assembly statistics for each Galloanserae species

Species	Number of sequenced reads	Number of mapped reads	Total sequence (Gb)	Ortholog assembly size (bp)	Percentage of sites in ortholog assembly passing SNP depth and quality filters (%)
Meleagris gallopavo	4.28×10^8	4.00×10^8	85.68	6,885,054	99.08
Phasianus colchicus	5.06×10^8	3.94×10^8	101.12	6,683,835	99.24
Numida meleagris	5.00×10^8	3.92×10^8	99.91	6,998,700	99.24
Anas platyrhynchos	5.01×10^8	3.97×10^8	100.23	7,030,167	99.05
Anser cygnoides	4.92×10^8	3.98×10^8	98.41	6,823,503	99.34
Pavo cristatus	4.63×10^8	3.72×10^8	92.65	6,996,528	98.94

Supplementary Table 2. Assembly statistics for each sample

Sample	Number of sequenced reads	Number of mapped reads	Average per base sequence quality for all reads post quality filtering (Phred score)
APLMG1	2.24×10^7	1.74×10^7	35.89
APLMG2	2.46×10^7	1.89×10^7	35.76
APLMG3	2.90×10^7	2.22×10^7	35.70
APLMG4	2.58×10^7	2.00×10^7	35.73
APLMG5	2.46×10^7	1.89×10^7	35.75
APLFG1	2.77×10^7	2.25×10^7	35.81
APLFG2	2.47×10^7	2.01×10^7	35.89
APLFG3	2.33×10^7	1.91×10^7	35.81
APLFG4	2.48×10^7	2.02×10^7	35.82
APLFG5	2.18×10^7	1.80×10^7	35.83
APLMS1	1.92×10^7	1.53×10^7	35.47
APLMS2	2.50×10^7	1.96×10^7	35.52
APLMS3	2.25×10^7	1.77×10^7	35.57
APLMS4	2.33×10^7	1.82×10^7	35.75
APLMS5	2.43×10^7	1.92×10^7	35.68
APLFS1	2.61×10^7	2.08×10^7	35.55
APLFS2	2.46×10^7	1.97×10^7	35.47
APLFS3	2.30×10^7	1.84×10^7	35.54
APLFS4	2.76×10^7	2.18×10^7	35.54
APLFS5	3.70×10^7	2.93×10^7	35.49
ACYMG1	2.69×10^7	2.04×10^7	35.78
ACYMG2	2.34×10^7	1.81×10^7	35.72
ACYMG3	2.49×10^7	1.95×10^7	35.72
ACYMG4	2.26×10^7	1.73×10^7	35.74
ACYMG5	2.33×10^7	1.81×10^7	35.72
ACYFG1	2.79×10^7	2.31×10^7	35.72
ACYFG2	2.44×10^7	2.02×10^7	35.62
ACYFG3	2.59×10^7	2.15×10^7	35.72
ACYFG4	2.72×10^7	2.24×10^7	35.38
ACYFG5	2.60×10^7	2.16×10^7	35.44
ACYMS1	2.21×10^7	1.85×10^7	35.23
ACYMS2	2.49×10^7	2.02×10^7	35.26
ACYMS3	2.19×10^7	1.78×10^7	35.30
ACYMS4	2.55×10^7	2.08×10^7	35.23
ACYMS5	2.56×10^7	2.10×10^7	35.14
ACYFS1	2.52×10^7	2.08×10^7	35.75
ACYFS2	2.65×10^7	2.17×10^7	35.74
ACYFS3	2.24×10^7	1.83×10^7	35.77
ACYFS4	2.01×10^7	1.62×10^7	35.75
ACYFS5	2.55×10^7	2.06×10^7	35.24
MGAMG1	2.43×10^7	1.89×10^7	35.40
MGAMG2	2.67×10^7	2.06×10^7	35.31
MGAMG3	2.61×10^7	2.01×10^7	35.25
MGAMG4	2.45×10^7	1.92×10^7	35.31
MGAMG5	3.03×10^7	2.31×10^7	35.28
MGAFG1	2.85×10^7	2.36×10^7	35.38
MGAFG2	2.87×10^7	2.37×10^7	35.41
MGAFG3	3.06×10^7	2.52×10^7	35.31
MGAFG4	2.64×10^7	2.20×10^7	35.36
MGAFG5	2.71×10^7	2.24×10^7	35.32
MGAMS1	2.32×10^7	1.80×10^7	35.56
MGAMS2	2.56×10^7	2.00×10^7	35.58
MGAMS3	2.95×10^7	2.32×10^7	35.14
MGAMS4	2.59×10^7	1.96×10^7	35.10
MGAFS1	2.68×10^7	2.09×10^7	35.55
MGAFS2	2.40×10^7	1.90×10^7	35.62
NMEMG1	2.56×10^7	1.97×10^7	35.65

NMEMG2	2.69×10^{7}	2.06×10^{7}	35.88
NMEMG3	2.21×10^{7}	1.68×10^{7}	35.82
NMEMG4	2.16×10^{7}	1.64×10^{7}	35.84
NMEMG5	2.18×10^{7}	1.67×10^{7}	35.89
NMEFG1	2.61×10^{7}	2.13×10^{7}	35.69
NMEFG2	2.87×10^{7}	2.35×10^{7}	35.69
NMEFG3	2.52×10^{7}	2.06×10^{7}	35.70
NMEFG4	2.29×10^{7}	1.86×10^{7}	35.70
NMEFG5	2.33×10^{7}	1.88×10^{7}	35.67
NMEMS1	2.25×10^{7}	1.73×10^{7}	35.35
NMEMS2	2.61×10^{7}	2.03×10^{7}	35.35
NMEMS3	2.42×10^{7}	1.88×10^{7}	35.35
NMEMS4	2.41×10^{7}	1.85×10^{7}	35.58
NMEMS5	2.28×10^{7}	1.78×10^{7}	35.53
NMEFS1	2.08×10^{7}	1.62×10^{7}	35.39
NMEFS2	2.29×10^{7}	1.81×10^{7}	35.30
NMEFS3	3.28×10^{7}	2.59×10^{7}	35.32
NMEFS4	2.99×10^{7}	2.36×10^{7}	35.34
NMEFS5	2.91×10^{7}	2.28×10^{7}	35.29
PCRMG1	1.99×10^{7}	1.56×10^{7}	35.70
PCRMG2	2.36×10^{7}	1.81×10^{7}	35.57
PCRMG3	2.15×10^{7}	1.68×10^{7}	35.56
PCRMG4	2.34×10^{7}	1.81×10^{7}	35.57
PCRMG5	2.03×10^{7}	1.59×10^{7}	35.64
PCRFG1	2.29×10^{7}	1.94×10^{7}	35.71
PCRFG2	2.60×10^{7}	2.19×10^{7}	35.71
PCRFG3	1.86×10^{7}	1.55×10^{7}	35.69
PCRFG4	3.43×10^{7}	2.88×10^{7}	35.67
PCRFG5	2.11×10^{7}	1.75×10^{7}	35.65
PCRMS1	2.14×10^{7}	1.71×10^{7}	35.63
PCRMS2	2.19×10^{7}	1.75×10^{7}	35.69
PCRMS3	2.54×10^{7}	2.05×10^{7}	35.56
PCRMS4	2.00×10^{7}	1.59×10^{7}	35.63
PCRMS5	2.20×10^{7}	1.77×10^{7}	35.51
PCRFS1	2.81×10^{7}	2.27×10^{7}	35.67
PCRFS2	1.98×10^{7}	1.62×10^{7}	35.57
PCRFS3	2.17×10^{7}	1.73×10^{7}	35.64
PCRFS4	3.56×10^{7}	2.81×10^{7}	35.59
PCRFS5	1.58×10^{7}	1.26×10^{7}	35.63
PCOMG1	2.41×10^{7}	1.86×10^{7}	35.62
PCOMG2	2.90×10^{7}	2.25×10^{7}	35.63
PCOMG3	2.28×10^{7}	1.80×10^{7}	35.49
PCOMG4	2.41×10^{7}	1.84×10^{7}	35.48
PCOMG5	2.06×10^{7}	1.62×10^{7}	35.50
PCOMG6	2.06×10^{7}	1.56×10^{7}	35.49
PCOFG1	2.28×10^{7}	1.87×10^{7}	35.66
PCOFG2	2.58×10^{7}	2.10×10^{7}	35.54
PCOFG3	2.43×10^{7}	1.98×10^{7}	35.54
PCOFG4	2.07×10^{7}	1.68×10^{7}	35.51
PCOFG5	2.10×10^{7}	1.68×10^{7}	35.41
PCOMS1	2.13×10^{7}	1.57×10^{7}	35.46
PCOMS2	2.48×10^{7}	1.83×10^{7}	35.41
PCOMS3	2.47×10^{7}	1.93×10^{7}	35.35
PCOMS4	2.53×10^{7}	1.97×10^{7}	35.51
PCOMS5	2.15×10^{7}	1.64×10^{7}	35.36
PCOMS6	2.45×10^{7}	1.83×10^{7}	35.37
PCOFS1	2.02×10^{7}	1.66×10^{7}	35.67
PCOFS2	2.28×10^{7}	1.67×10^{7}	35.57
PCOFS3	2.17×10^{7}	1.66×10^{7}	35.64
PCOFS4	2.12×10^{7}	1.69×10^{7}	35.59
PCOFS5	2.19×10^{7}	1.70×10^{7}	35.63

APL, ACY, MGA, NME, PCR and PCO refers to *A. platyrhynchos, A. cygnoides, M. gallopavo, N. meleagris, P. cristatus and P. colchicus.*
M, F, G and S refers to male, female, gonad and spleen samples.

Supplementary Table 3. Number of nonsynonymous and synonymous polymorphic and fixed sites at a minor allele frequency threshold of 0.15

Species	Z chromosome					Autosomes 1-10				
	D_N	D_S	P_N	All P_S	P_{4D}	D_N	D_S	P_N	All P_S	P_{4D}
Meleagris gallopavo	4472	11563	51	83	58	38835	118922	1174	3276	1936
Phasianus colchicus	4094	11419	89	157	105	37842	117996	1654	4950	3035
Numida meleagris	3565	9472	29	100	56	33399	99449	1339	3737	2322
Anas platyrhynchos	2901	8233	126	351	204	26162	87629	2417	9542	5927
Anser cygnoides	2379	7093	127	206	134	22486	74388	2138	5657	3498
Pavo cristatus	3750	10420	38	63	36	35767	110749	610	1301	769

4D refers to fourfold degenerate sites.

Supplementary Table 4. Number of nonsynonymous and synonymous polymorphic and fixed sites at a minor allele frequency threshold of 0.25

Species	Z chromosome					Autosomes 1-10				
	D_N	D_S	P_N	All P_S	P_{4D}	D_N	D_S	P_N	All P_S	P_{4D}
Meleagris gallopavo	4472	11563	22	37	27	38835	118922	595	1764	1033
Phasianus colchicus	4094	11419	27	38	24	37842	117996	759	2289	1394
Numida meleagris	3565	9472	8	16	11	33399	99449	697	1959	1232
Anas platyrhynchos	2901	8233	44	117	67	26162	87629	1112	4753	2977
Anser cygnoides	2379	7093	38	76	52	22486	74388	1329	3696	2292
Pavo cristatus	3750	10420	9	9	5	35767	110749	297	626	373

4D refers to fourfold degenerate sites.

Supplementary Table 5. Phylogenetically controlled regression analyses between Faster-Z and log sperm number, and residual testes weight

Species excluded	Residual testes weight				Log sperm number			
	t_4	p	r^2	Beta	t_4	p	r^2	Beta
Meleagris gallopavo	3.606	**0.011**	0.711	0.024	5.807	**0.002**	0.863	0.017
Phasianus colchicus	6.300	**0.002**	0.887	0.037	3.715	**0.010**	0.730	0.021
Numida meleagris	2.542	**0.032**	0.559	0.033	3.300	**0.015**	0.683	0.021
Anas platyrhynchos	1.328	0.127	0.261	0.027	2.283	**0.042**	0.509	0.028
Anser cygnoides	1.490	0.105	0.307	0.031	9.730	**<0.001**	0.950	0.060
Pavo cristatus	2.329	**0.040**	0.514	0.030	3.571	**0.012**	0.715	0.021

Parameters are shown for the regression analysis when the given species is excluded.
Bold values indicate that the slope is significantly greater than 0.

Supplementary Table 6. Nucleotide diversity estimates of the Z chromosome and autosomes

Species	Z chromosome				Autosomes 1-10			
	θ_{4D}	π_{4D}	θ_S	π_S	θ_{4D}	π_{4D}	θ_S	π_S
Meleagris gallopavo	<0.000	0.002	<0.000	0.001	0.001	0.005	0.001	0.005
Phasianus colchicus	0.001	0.003	0.001	0.002	0.002	0.008	0.002	0.007
Numida meleagris	<0.000	0.001	<0.000	0.002	0.002	0.006	0.002	0.005
Anas platyrhynchos	0.002	0.005	0.002	0.005	0.004	0.015	0.004	0.014
Anser cygnoides	0.001	0.004	0.001	0.003	0.002	0.009	0.002	0.008
Pavo cristatus	<0.000	0.001	<0.000	0.001	0.001	0.002	0.002	0.002

Minor allele frequency threshold of 0.15.
4D refers to diversity estimates calculated using four-fold degenerate sites and S to all synonymous sites.

Supplementary Table 7. Effective population size estimates of the Z chromosome and autosomes

Species	N_{EZ} (E+05) (95% CI)		$N_{EA1\text{-}10}$ (E+05) (95% CI)		$N_{EZ}/N_{EA1\text{-}10}$ (95% CI)	
	4D	S	4D	S	4D	S
Meleagris gallopavo	1.761 (1.0874-2.702)	1.497 (0.958-2.172)	6.047 (5.656-6.469)	6.044 (5.703-6.425)	0.291 (0.179-0.426)	0.248 (0.167-0.364)
Phasianus colchicus	3.188 (2.308-4.210)	2.831 (2.056-3.816)	9.481 (8.948-10.054)	9.133 (8.724-9.555)	0.336 (0.234-0.460)	0.310 (0.219-0.420)
Numida meleagris	1.695 (0.773-3.213)	1.801 (0.745-3.295)	7.233 (6.682-7.848)	6.887 (6.401-7.397)	0.234 (0.103-0.423)	0.261 (0.108-0.494)
Anas platyrhynchos	6.150 (3.927-8.758)	6.307 (4.233-8.975)	18.427 (17.447-19.544)	17.571 (16.735-18.510)	0.334 (0.209-0.470)	0.359 (0.242-0.511)
Anser cygnoides	4.045 (2.774-5.591)	3.704 (2.467-5.165)	10.894 (10.233-11.570)	10.425 (9.869-10.980)	0.371 (0.250-0.529)	0.355 (0.240-0.500)
Pavo cristatus	1.088 (0.167-2.811)	1.133 (2.189-2.719)	2.393 (2.095-2.697)	2.397 (2.131-2.698)	0.455 (0.057-1.227)	0.473 (0.092-1.177)

$N_E = \pi/(4*(U*\text{generation time}))$.

Mutation rate estimates are from Axelsson et al. 2004, Dimcheff et al. 2002 and van Tuinen and Dyke 2004.

Minor allele frequency threshold of 0.15.

4D refers to N_E calculated using four-fold degenerate sites and S to all synonymous sites.

Supplementary Table 8. Effective population size estimates of the Z chromosome and autosomes calculated using Watterson's estimation of theta

Species	N_{EZ} (E+05) (95% CI)		N_{EA1-10} (E+05) (95% CI)		N_{EZ}/N_{EA1-10} (95% CI)	
	4D	S	4D	S	4D	S
Meleagris gallopavo	0.542 (0.347-7.978)	0.460 (0.292-0.690)	1.705 (1.587-1.819)	1.703 (1.603-1.818)	0.318 (0.199-0.490)	0.270 (0.170-0.405)
Phasianus colchicus	0.943 (0.658-1.244)	0.837 (0.604-1.113)	2.601 (2.461-2.742)	2.506 (2.393-2.619)	0.362 (0.265-0.503)	0.334 (0.246-0.450)
Numida meleagris	0.521 (2.385-9.539)	0.554 (0.230-1.000)	2.038 (1.883-2.220)	1.941 (1.796-2.096)	0.256 (0.113-0.464)	0.285 (0.116-0.525)
Anas platyrhynchos	1.892 (1.196-2.686)	1.939 (1.277-2.745)	5.195 (4.880-5.525)	4.952 (4.704-5.225)	0.364 (0.232-0.534)	0.392 (0.254-0.565)
Anser cygnoides	1.244 (0.840-1.778)	1.139 (0.768-1.549)	3.072 (2.885-3.272)	2.938 (2.783-3.098)	0.405 (0.258-0.565)	0.388 (0.269-0.526)
Pavo cristatus	0.335 (0.048-0.899)	0.348 (0.071-0.867)	0.675 (0.591-0.756)	0.675 (0.606-0.768)	0.496 (0.073-1.356)	0.516 (0.105-1.305)

$N_E = \theta/(4*(U*\text{generation time}))$.
Mutation rate estimates are from Axelsson et al. 2004, Dimcheff et al. 2002 and van Tuinen and Dyke 2004.
Minor allele frequency threshold of 0.15.
4D refers to N_E calculated using four-fold degenerate sites and S to all synonymous sites.

Supplementary Table 9. Phylogenetically controlled regression analyses between different measures of N_{EZ}/N_{EA} and residual testes weight, log sperm number, and Faster-Z

N_E method	Residual testes weight				Log sperm number				Faster-Z			
	t_4	p	r^2	Beta	t_4	p	r^2	Beta	t_4	p	r^2	Beta
π_{4D}	0.656	0.275	0.068	-0.020	0.735	0.252	0.083	-0.014	1.300	0.132	0.220	-0.259
π_S	0.599	0.292	0.058	-0.022	0.411	0.352	0.028	-0.009	1.243	0.143	0.205	-0.213
θ_{4D}	0.681	0.268	0.073	0.681	0.734	0.252	0.083	-0.015	1.268	0.137	0.211	-0.233
θ_S	0.617	0.287	0.061	-0.024	0.409	0.352	0.028	-0.010	1.221	0.146	0.200	-0.192

$N_E = \theta/(4*(U*\text{generation time}))$ or $N_E = \pi/(4*(U*\text{generation time}))$.
4D refers to N_E calculated using four-fold degenerate sites and S to all synonymous sites.

Supplementary Table 10. Site model test results for contigs with signatures of positive selection

G.gallus ortholog [a]	Chromosome	ω	Proportion of sites	M1a likelihood ratio	M2a likelihood ratio	LRT	p-value	p-fdr value [b]
22552	1	2.897	0.122	-6535.857	-6522.227	27.259	**< 0.001**	**0.003**
21101	1	4.155	0.033	-14063.297	-14050.286	26.023	**< 0.001**	**0.006**
37104	1	4.926	0.036	-4435.291	-4424.814	20.954	**< 0.001**	0.073
36845	1	4.415	0.075	-3419.672	-3409.907	19.531	**< 0.001**	0.148
16502	1	3.237	0.144	-2264.272	-2255.709	17.126	**< 0.001**	0.492
21418	1	3.079	0.055	-5553.341	-5545.776	15.130	**0.001**	1.334
21457	1	1.954	0.069	-12752.825	-12745.364	14.922	**0.001**	1.479
24837	1	3.717	0.093	-1309.612	-1303.078	13.069	**0.001**	3.731
23319	1	5.404	0.008	-11327.088	-11320.745	12.686	**0.002**	4.515
14357	1	2.471	0.174	-3251.079	-3244.950	12.258	**0.002**	5.588
26993	1	20.333	0.008	-1343.505	-1338.433	10.145	**0.006**	16.008
23629	1	3.562	0.044	-3033.798	-3029.348	8.900	**0.012**	29.785
27590	1	5.082	0.013	-6679.670	-6675.904	7.531	**0.023**	58.805
26389	1	20.985	0.002	-2662.400	-2659.000	6.800	**0.033**	84.670
27463	1	4.641	0.021	-2788.178	-2784.963	6.429	**0.040**	101.587
44252	2	2.831	0.165	-1432.129	-1427.436	9.386	**0.009**	23.370
45482	2	10.290	0.002	-4950.364	-4946.989	6.749	**0.034**	86.841
26112	2	1.687	0.167	-5851.565	-5848.226	6.677	**0.035**	89.889
46085	2	1.705	0.326	-2701.980	-2698.729	6.502	**0.039**	98.009
20347	2	1.705	0.099	-9450.060	-9446.902	6.315	**0.043**	107.477
31776	3	4.608	0.130	-1270.098	-1256.430	27.337	**< 0.001**	**0.003**
14587	3	4.393	0.040	-2761.834	-2753.484	16.700	**< 0.001**	0.609
16201	3	3.925	0.027	-8837.436	-8829.793	15.286	**< 0.001**	1.235
24568	3	5.232	0.015	-4629.778	-4625.429	8.697	**0.013**	32.942
17328	3	2.585	0.112	-1886.600	-1883.057	7.085	**0.029**	73.470
37447	3	2.523	0.078	-1740.906	-1737.739	6.333	**0.042**	106.550
16068	3	6.676	0.006	-2283.082	-2280.007	6.150	**0.046**	116.661
17992	4	3.812	0.033	-10248.079	-10239.377	17.406	**< 0.001**	0.428
23095	4	4.149	0.023	-10276.768	-10269.900	13.737	**0.001**	2.674
22955	4	4.580	0.013	-9871.493	-9864.857	13.274	**0.001**	3.368
14387	4	6.044	0.014	-5452.766	-5447.180	11.173	**0.004**	9.600
32094	4	4.363	0.038	-2032.598	-2028.562	8.072	**0.018**	44.952
22858	4	21.691	0.003	-3252.065	-3248.231	7.668	**0.022**	54.956
25298	4	3.750	0.055	-2108.140	-2104.827	6.626	**0.036**	92.137
12746	5	3.833	0.042	-3266.303	-3260.081	12.445	**0.002**	5.090
12190	5	7.557	0.007	-7413.128	-7407.132	11.992	**0.002**	6.382
17502	5	4.310	0.012	-9231.539	-9225.994	11.091	**0.004**	9.996
18863	5	1.521	0.177	-10833.724	-10829.701	8.046	**0.018**	45.523
14067	5	4.615	0.004	-5474.941	-5471.907	6.067	**0.048**	121.549
39919	6	4.226	0.310	-1630.735	-1611.278	38.915	**< 0.001**	**< 0.001**
10311	6	6.691	0.043	-1636.382	-1626.707	19.351	**< 0.001**	0.162
13711	6	2.190	0.129	-7523.106	-7517.618	10.975	**0.004**	10.580

06741	7	5.639	0.020	-4454.582	-4447.832	13.499	**0.001**	3.011
18021	7	2.755	0.046	-8843.415	-8837.885	11.060	**0.004**	10.147
21611	7	3.740	0.024	-7320.455	-7316.242	8.426	**0.015**	37.692
14983	7	26.827	0.002	-3520.059	-3515.955	8.207	**0.017**	42.024
03831	8	4.817	0.080	-9607.226	-9560.287	93.878	**< 0.001**	**< 0.001**
09004	8	8.990	0.007	-4487.428	-4483.521	7.813	**0.020**	51.111
00626	8	2.660	0.029	-7503.872	-7500.522	6.700	**0.035**	88.907
013645	9	6.164	0.005	-4614.796	-4610.506	8.580	**0.014**	34.924
11804	10	4.057	0.006	-3908.867	-3903.212	11.309	**0.004**	8.972
39713	11	2.279	0.254	-2696.490	-2691.001	10.980	**0.004**	10.559
41379	11	2.222	0.330	-974.822	-971.467	6.711	**0.035**	88.436
01593	11	3.991	0.061	-1953.134	-1949.807	6.654	**0.036**	90.900
44152	11	8.060	0.020	-1087.697	-1084.699	5.997	**0.050**	125.827
34137	12	1.757	0.408	-3027.514	-3022.796	9.435	**0.009**	22.810
10504	15	3.343	0.072	-5389.616	-5375.473	28.287	**< 0.001**	**0.002**
11333	17	3.164	0.041	-7288.959	-7283.483	10.952	**0.004**	10.699
05557	18	3.172	0.050	-6000.642	-5995.829	9.626	**0.008**	20.744
02792	19	4.005	0.038	-1981.892	-1971.930	19.923	**< 0.001**	0.122
01367	19	25.280	0.001	-5913.302	-5909.030	8.544	**0.014**	35.534
01868	20	9.422	0.013	-4192.958	-4179.195	27.526	**< 0.001**	**0.003**
07089	21	2.003	0.294	-2347.262	-2343.741	7.042	**0.030**	75.049
02022	28	4.914	0.068	-2768.690	-2753.634	30.110	**< 0.001**	**0.001**
29351	Z	6.802	0.104	-2974.892	-2930.430	88.925	**< 0.001**	**< 0.001**
24394	Z	4.087	0.029	-6594.462	-6587.094	14.736	**0.001**	1.624
23931	Z	9.163	0.007	-3248.608	-3242.892	11.431	**0.003**	8.443
08032	Z	13.117	0.001	-8849.748	-8844.406	10.685	**0.005**	12.226
24368	Z	5.886	0.011	-7464.550	-7461.493	6.113	**0.047**	118.812

[a] ENSGALT000000…..
[b] Sequential Bonferroni correction (Holm 1979).

Supplementary Table 11. p_N, p_S and p_N/p_S for Z-linked and autosomal genes across Galloanserae species with a minor allele frequency of 0.25

Species	Z chromosome			Autosomes 1-10			Faster-Z Effect
	p_N (95% CI)	p_S (95% CI)	p_N/p_S (95% CI)	p_N (95% CI)	p_S (95% CI)	p_N/p_S (95% CI)	$p_{NZ}/p_{SZ} : p_{NA}/p_{SA}$ (95% CI)
Meleagris gallopavo	0.000 (0.000-0.000)	0.001 (0.000-0.001)	0.170 (0.081-0.285)	0.000 (0.000-0.000) p = 1.000	0.003 (0.002-0.003) p = 1.000	**0.096 (0.085-0.108) p < 0.001**	1.770 (0.891-2.971)
Phasianus colchicus	0.000 (0.000-0.000)	0.001 (0.000-0.001)	0.203 (0.093-0.362)	0.000 (0.000-0.000) p = 1.000	0.003 (0.003-0.004) p = 1.000	**0.094 (0.086-0.104) p < 0.001**	2.152 (0.982-4.002)
Numida meleagris	0.000 (0.000-0.000)	0.000 (0.000-0.000)	0.143 (0.040-0.407)	0.000 (0.000-0.000) p = 1.000	0.003 (0.003-0.003) p = 1.000	**0.101 (0.089-0.113) p = 0.031**	1.412 (0.326-4.247)
Anas platyrhynchos	0.000 (0.000-0.000)	0.002 (0.001-0.003)	0.108 (0.054-0.205)	0.000 (0.000-0.001) p = 1.000	0.007 (0.007-0.007) p = 1.000	**0.067 (0.060-0.074) p = 0.001**	1.618 (0.818-3.120)
Anser cygnoides	0.000 (0.000-0.000)	0.001 (0.001-0.002)	0.144 (0.070-0.231)	0.001 (0.000-0.001) p = 1.000	0.005 (0.005-0.006) p = 1.000	**0.103 (0.094-0.113) p = 0.005**	1.400 (0.674-2.315)
Pavo cristatus [a]	0.000 -	0.000 -	0.287 -	0.000 (0.000-0.000) p = 0.997	0.001 (0.001-0.001) p = 1.000	**0.135 (0.110-0.163) p = 0.002**	2.119 -

[a] Too few Z-linked polymorphisms to conduct bootstrapping for *P. cristatus* (Supplementary Table 4)

Supplementary Table 12. McDonald Kreitman test results

Species	Z chromosome		Autosomes 1-10	
	No. of genes [a]	Positive selection [b/c]	No. of genes	Positive selection
Meleagris gallopavo	3	0/0	254	1/0
Phasianus colchicus	12	0/0	378	2/0
Numida meleagris	4	0/0	258	1/1
Anas platyrhynchos	17	0/0	581	8/0
Anser cygnoides	15	0/0	426	3/0
Pavo cristatus	3	0/0	83	1/0
**Total**	**54**	**0/0**	**1980**	**16/1**

[a] Contigs were excluded if the sum of each marginal row and column of the 2x2 contingency table was less than 6.
[b] p-values were corrected with a false discovery rate of 0.05 and lambda 0 to obtain q-values. The number of contigs with a significant uncorrected p-value is shown first, followed by the number after correction for multiple testing.
[c] Excess of nonsynonymous substitutions relative to polymorphism indicates positive selection.
Minor allele frequency threshold of 0.15.

Supplementary Table 13. Significant differences between nonsynonymous and synonymous polymorphism on the Z chromosome and autosomes with a minor allele frequency threshold of 0.25

Species	Z chromosome		Autosomes 1-10		Faster-Z Effect
	P_N	P_S	P_N	P_S	$P_{NZ/}\ P_{SZ} : P_{NA/}\ P_{SA}$ p-value
Meleagris gallopavo	22	37	595	1764	1.763<br>$p = 0.051$
Phasianus colchicus	27	38	759	2289	**2.143**<br>**$p = 0.004$**
Numida meleagris	8	16	697	1959	1.405<br>$p = 0.581$
Anas platyrhynchos	44	117	1112	4753	**1.607**<br>**$p = 0.010$**
Anser cygnoides	38	76	1329	3696	1.391<br>$p = 0.124$
Pavo cristatus	9	9	297	626	2.108<br>$p = 0.179$

Significant differences were determined using Pearson's Chi-squared test in R.
Significant differences between autosomal and Z-linked orthogroups are shown in bold.
Minor allele frequency threshold of 0.25.

Supplementary Table 14. Faster-Z Effect across expression classes

Species	Female-biased $d_{NZ}/d_{SZ} : d_{NA}/d_{SA}$ 95% CI	Unbiased $d_{NZ}/d_{SZ} : d_{NA}/d_{SA}$ 95% CI	Male-biased $d_{NZ}/d_{SZ} : d_{NA}/d_{SA}$ 95% CI
Meleagris gallopavo	1.250 (0.772-1.883) Z = 24, A = 394	1.104 (0.874-1.325) Z = 73, A = 853	1.217 (0.907-1.560) Z = 63, A = 438
Phasianus colchicus	1.091 (0.639-1.619) Z = 21, A = 419	1.015 (0.812-1.253) Z = 77, A = 863	1.199 (0.897-1.525) Z = 60, A = 404
Numida meleagris	1.068 (0.754-1.451) Z = 26, A = 402	0.827 (0.659-1.011) Z = 75, A = 897	1.490 (1.157-1.901) Z = 58, A = 381
Anas platyrhynchos	1.026 (0.691-1.449) Z = 45, A = 454	1.141 (0.833-1.536) Z = 65, A = 828	1.350 (0.920-1.828) Z = 50, A = 400
Anser cygnoides	1.146 (0.735-1.706) Z = 24, A = 413	0.867 (0.659-1.104) Z = 74, A = 835	1.434 (1.038-1.862) Z = 61, A = 426
Pavo cristatus	1.289 (0.942-1.725) Z = 27, A = 412	0.912 (0.704-1.135) Z = 64, A =803	1.245 (0.930-1.619) Z = 69, A = 461

Expression category was assigned using both fold change and t-tests separately for each species.
A refers to autosomes 1-10.

Supplementary Table 15. Differences in Faster-Z Effect between expression classes

Species	Female- and male-biased p-value/fdr p-value	Female- and unbiased p-value/fdr p-value	Male- and unbiased p-value/fdr p-value
Meleagris gallopavo	0.870/1.740	0.548/3.836	0.590/3.540
Phasianus colchicus	0.782/2.346	0.736/2.944	0.376/3.760
Numida meleagris	0.194/2.716	0.224/2.912	**<0.001/<0.020**
Anas platyrhynchos	0.346/3.806	0.732/3.660	0.496/3.968
Anser cygnoides	0.444/3.996	0.272/3.264	**0.010**/0.170
Pavo cristatus	0.894/0.894	0.100/1.600	0.138/2.070

Expression category is assigned using both fold change and t-tests separately for each species. Significant differences in Faster-Z Effect between expression categories are shown in bold and calculated using 1000 permutation tests.
P-values corrected for multiple tests using sequential Bonferroni correction.
